# Assessment of a taping method combined with manual therapy as a treatment of non-specific chronic low back pain – a randomized controlled trial

**DOI:** 10.1186/s12891-021-04236-2

**Published:** 2021-05-04

**Authors:** Stefan Schmidt, Nicolas Wölfle, Claudia Schultz, Dieter Sielmann, Roman Huber, Harald Walach

**Affiliations:** 1 Department for Psychosomatic Medicine and Psychotherapy, Medical Faculty, Medical Center – University of Freiburg, Hauptstr. 8, 79104 Freiburg, Germany; 2 Institute for Frontier Areas of Psychology and Mental Health, Freiburg, Germany; 3 University Centre for Complementary Medicine, Medical Faculty, Medical Center – University of Freiburg, Freiburg, Germany; 4 GP Practice B80, Gundelfingen, Germany; 5 GP practice, Bad Oldesloe, Germany; 6 Pediatric Clinic, Medical University Poznan, Poznan, Poland; 7 Department of Psychology and Psychotherapy, University Witten-Herdecke, Witten, Germany; 8 CHS Institute, Berlin, Germany

**Keywords:** Taping, Chronic low back pain, Manual therapy, RCT, Leg length difference

## Abstract

**Background:**

Chronic low back pain is the most frequent medical problem and the condition with the most years lived with disability in Western countries. The objective of this study was to assess a new treatment, Medi-Taping, which aims at reducing complaints by treating pelvic obliquity with a combination of manual treatment of trigger points and kinesio taping in a pragmatic RCT with pilot character.

**Methods:**

One hundred ten patients were randomized at two study centers either to Medi-Taping or to a standard treatment consisting of patient education and physiotherapy as control. Treatment duration was 3 weeks. Measures were taken at baseline, end of treatment and at follow-up after 2 months. Main outcome criteria were low back pain measured with VAS, the Chronic Pain Grade Scale (CPGS) and the Oswestry Low Back Pain Disability Questionnaire (ODQ).

**Results:**

Patients of both groups benefited from the treatment by medium to large effect sizes. All effects were pointing towards the intended direction. While Medi-Taping showed slightly better improvement rates, there were no significant differences for the primary endpoints between groups at the end of treatment (VAS: mean difference in change 0.38, 95-CI [− 0.45; 1.21] *p* = 0.10; ODQ 2.35 [− 0.77; 5.48] *p* = 0.14; CPGS − 0.19 [− 0.46; 0.08] *p* = 0.64) and at follow-up. Health-related quality of life was significantly higher (*p* = .004) in patients receiving Medi-Taping compared to controls.

**Conclusions:**

Medi-Taping, a purported way of correcting pelvic obliquity and chronic tension resulting from it, is a treatment modality similar in effectiveness to complex physiotherapy and patient education.

**Trial registration:**

This trial was registered retrospectively on July 24th, 2019 as Number DRKS00017051 in the German Register of Clinical Trials (Deutsches Register Klinischer Studien).

URL of trial registry record: https://www.drks.de/drks_web/navigate.do?navigationId=trial.HTML&TRIAL_ID=DRKS00017051.

## Background

Chronic low back pain (CLBP) is one of the most frequent medical problems in Western countries. Lifetime prevalence rates of about 75% and point prevalence rates between 32 and 49% have been documented by epidemiological studies in Germany [1, 2] and are similar elsewhere in Europe [3–5]. Worldwide, it is the condition with the most years lived with disability [6]. Although we have powerful medications to treat acute pain conditions, for chronic conditions most guidelines advise against medications, because of side-effects in long-term users even of simple nonsteroidal anti-inflammatory substances, because of a lack of clinical effectiveness and because of potential dependency problems with stronger opiates [7–10]. Thus, chronic low back pain still poses a therapeutic challenge to general practitioners, who are the first in line for patients afflicted with the condition. Studies on physiotherapy (including mobilization) as well as exercise, Yoga, Tai Chi and other forms of complex modalities have all shown some efficacy for CLBP [11–15], yet some patients either remain unimproved even after complex applications or standard therapies, or are unwilling to undergo complex and comparatively demanding therapeutic regimes such as yoga therapy or exercise programs.

The etiology of CLBP is unclear in most cases. We know in general terms that in some patients central hypersensitization as well as pain memory on the level of the spinal cord ganglia can play a role. But in most cases, many factors contribute to generating and sustaining a chronic pain problem [16]. Local or distal tensions are likely contributors, and ultrasound data point to the fact that connective tissue alterations might be the cause for pain [17]. Such connective tissue alterations are likely the long term-sequelae of past trauma [18] or chronic postural or functional muscular problems [19–23]. Specifically postural problems such as pelvic obliquity are a neglected potential cause. This is an under-researched area with very few studies documenting it [24].

This was one of the starting points for our therapeutic rationale: In the clinical experience of one of the authors (DS) pelvic obliquity might be a potential co-factor in a multicausal network of factors contributing to the genesis of CLBP, resulting in chronic tension and painful trigger points that radiate out or induce pain through secondary reflex systems such as the Head zones [20, 21]. Thus, he developed a method of treating CLBP using a certain kind of kinesio tape, but in a modified context. Classical kinesio tape was developed by Kenzo Kase in the 1960s, mainly to treat and prevent sports injuries [25]. It has become a standard treatment in sports medicine, despite a lack of clear evidence of its clinical effectiveness [26–31]. With respect to the effects of kinesio taping on CLBP specifically, three recent systematic reviews report mixed results. While one review shows rather large effects regarding pain experience and functional disability [32], the other two could not report any advantage of kinesio tape [33, 34]. It may be important to mention that all reviews show large heterogeneity and comprise only a small set of 8–11 studies with a limited number of patients.

The method of using kinesio tape in our study is contingent on DS’s model of pelvic obliquity and secondary trigger points. Thus, in our treatment model those trigger points are treated by acupressure and massage first and the muscular reflex patterns are disrupted by placing kinesio tape along those muscles that are thought to be responsible for triggering and supporting the pain processes. This combined treatment approach is termed *Medi-Taping*. So far, there are no studies assessing the effectiveness or efficacy of this approach.

We designed this pragmatic trial in order to evaluate whether this particular type of kinesio taping used as a supportive treatment for correcting pelvic obliquity (a potential cofactor in causing CLBP) warrants further research. To the best of our knowledge, this is the first RCT assessing the effect of kinesio taping used in this way. Consequently, our trial has some characteristics of a pilot study, although its main objective is to assess clinical efficacy.

More specifically, our trial assessed the clinical effects of correcting pelvic obliquity and trigger points with short acupressure and massage and applying kinesio tape to the respective muscles. We compared this new modality to best-practice physiotherapy, a strong control as its use as a treatment is supported by guidelines for treating CLBP due to evidence of effectiveness [35, 36]. Since we did not know what effect sizes were to be expected for the Medi-Taping condition and in what domains potential effects would show, we opted for a broad array of pain and functional measurements following respective recommendations [37]. Since this was a first study, we defined several primary and secondary outcomes. Based on the clinical experience of DS in his GP clinic, we opted for a superiority design.

## Methods

### Design

We conducted a pragmatic, assessor-blinded, randomized controlled trial at two different study centers in Germany. Patients with CLBP were randomly allocated to either Medi-Taping or a standard treatment for CLPB consisting of patient education and physiotherapy for 3 weeks. Measurements were taken at baseline (t_1_), after the end of the respective treatment (t_2_, 4 weeks after baseline) and at follow-up 2 months after the end of treatment (t_3,_ 3 months after baseline).

Patients were recruited by public information, i.e., a press release by the Medical Center of the University of Freiburg, newspaper articles, information on the intranet of the Medical Center, information leaflets in pharmacies, and radio interviews at each study center. Additionally, the GP clinic of DS in the study center Bad Oldesloe is well known for low back pain treatment and attracted many patients due to its good reputation.

One study center was the Outpatient Center for Complementary Medicine at the Medical Center of the University of Freiburg in the south of Germany (Study Center South). Patients allocated to Medi-Taping were treated within the outpatient center while patients randomized to physiotherapy were treated in a larger private physiotherapy center (Reha Süd GmbH, Freiburg, Germany). The second center was the clinic for general medicine of the author DS in Bad Odesloe in the northern part of Germany (Study Center North). DS is the physician who developed Medi-Taping method (see also Section Background). Here patients allocated to Medi-Taping were treated within the clinic of DS, patients receiving physiotherapy were sent to a physiotherapy center (Reha Aktiv, Bad Oldesloe, Germany).

The RCT was approved by the ethics committee of the Medical Center, University of Freiburg. All patients gave written informed consent before inclusion into the trial.

### Patients

Criteria for the inclusion in our trial were unspecific low back pain for more than 12 weeks, a rating of at least 4 cm on a 10 cm Visual Analog Scale (VAS) for the back pain, age between 18 and 80 years, and the ability to read and communicate in German.

Patients fulfilling any of the following criteria were excluded from the trial: neurological malfunction at the lower extremities related to CLBP; a rating larger than 8 cm on the VAS for back pain; back pain due to infection, tumor, osteoporosis, or stenosis of the cerebrospinal canal; slipped vertebra/e; vertebral fractures; spinal disc herniation; surgery of vertebra, spinal disc or sacroiliac joint; allergy to tape; pregnancy; current cancer diagnosis; addictive disorder; severe psychiatric disorder; significant impairment due to memory problems or brain disorders; artificial hip ankle or knee joint; participation in other RCTs.

### Intervention

All patients received an active treatment for 3 weeks. This was either Medi-Taping or a standard treatment of a combination of patient education and physiotherapy.

#### Medi-taping

Patients received three treatments within 3 weeks on average, i.e., one treatment per week. The procedure within a session was standardized and manualized. According to this protocol the sessions started with an assessment of leg length difference. Patients were asked to lie on their back and the legs were slightly stretched by a soft pull at the ankles. Next, a continuous horizontal line was drawn on the inside of both calves indicating the position of the calves relative to each other. Then the patient was asked to sit up with the legs remaining outstretched. This procedure results in a shift of the line between the two calves for most people. This shift was measured in millimeters as leg length difference.

The patient was then asked to stretch out, lying supine, and the therapist palpated any myogeloses (areas of abnormal hardening in a muscle) and tense muscles areas that could be found next to the cervical spine between the base of the skull and seventh cervical vertebra on both sides. After this treatment the leg length difference assessment was repeated. If there was still a substantial difference. The same treatment was also performed on the thoracic and lumbar spine. Also, the mandibular joint was assessed for tense muscles and, if necessary, treated by palpation.

Next, the leg length difference was assessed again and several tapes were applied as follows: First, two parallel tapes were fixed on both sides of the spine above the erector spinae muscles ranging from the base of the skull to the sacrum. For the application patients were asked to bend forward and to lean on a bench. This position stretches the back and its anatomical structure before applying the tape and thus provides the tape with tension before fixing it. Next a star-shaped pattern of tape (three stripes meeting in one point) was placed on the lower back while the patient was still in the same bent position. Thus, the star tape covered the area of the patient’s maximum pain and additionally stabilized the sacroiliac joint. This tape was placed with maximum tension in the middle section by stretching the tape before application, with the ends (approx. 5 cm) applied without tension. If after this procedure there was still residual pain, a third tape was placed at the gluteus maximus muscle. This tape was first fixed distally from the greater trochanter then stretched up to approx. 80% of the possible tension before the other end was placed on the sacrum. On average six tapes were applied for the gluteus tape.

Patients were instructed to keep the tapes on as long as they stuck to the skin. If the patients had recurring low back pain (LBP) within the same week, they were asked to see the therapist again immediately. Otherwise, the second and the third treatment were scheduled once a week for the following 2 weeks, respectively.

Overall, three therapists delivered the intervention: CS and NW at Freiburg and DS at Bad Oldeslohe. NW and CS received training and visited courses on the intervention by DS. Before the start of the study DS supervised the work of NW and CS with several patients in pilot sessions.

#### Standard treatment

Patients allocated to the control or standard group received a patient education booklet containing information for patients with low back pain. This 100 page booklet was published by the commission that produced the German national guidelines for the treatment of low back pain [38]. It gives general information on the spinal system and CLBP; recommends self-help strategies in daily life; reports on the respective medical examinations, treatment options, and professional groups working in the CLPB treatment; and gives sources for further information. Furthermore, they received standard physiotherapy for CLBP; 6 sessions of 20 min duration within 3 weeks. Physiotherapy was conducted by professional and commercial physiotherapy centers. They were paid for the treatment by the study center and provided standard documentation of the sessions conducted, but were not otherwise associated with the study.

### Outcome measures

The following outcome measures were applied.

#### VAS

Average low back pain within the previous 2 weeks was assessed by a Visual Analog Scale (VAS) of 10 cm length with the anchor points at 0 cm ‘no pain at all’ and at 10 cm ‘worst possible pain’ [39, 40]. A difference of 2 cm was taken as the criterion of minimum clinically important difference (MCID) [41].

#### Oswestry low back pain disability questionnaire

Functional limitations due to CLBP were assessed using the German version of the Oswestry Low Back Pain Disability Questionnaire (ODQ) [42, 43]. The ODQ is a self-completed questionnaire with ten items covering pain intensity, ability to care for oneself, lifting and carrying, ability to walk, ability to sit, ability to stand, sleep quality, social life, sexuality and ability to travel. Every item has six statements describing possible situations in the patient’s life. The most applicable statement is checked by the patient. Questions are scored on a scale of 0–5. We adapted the questionnaire by omitting one item regarding sexual function. The MCID for this scale is 10 points according to [44].

#### Chronic pain grade scale/Korff grading (CPGS)

This instrument consists of 6 numeric rating scales and a single question [45]. The first item asks about activities of daily living and on how many days these could not be performed. Items two to four ask about current pain, worst pain and median pain during the previous 3 months in a numeric format. Items five and six ask about the impact of pain on daily life and family/leisure activities over the previous 3 months. These items are transformed into a grading of pain severity from one to four, with grade 1 and 2 reflecting low and grade 3 and 4 high disability. Cronbach’s alpha was reported to be .74 and thus acceptable in the original version and .88 in the German language version [46].

#### Range of motion: fingertip-to-floor (FTF)

The distance between fingertips and floor (FTF) is a measure of the flexibility of the spine. It is measured by bending forward with stretched legs as far as possible. The distance between fingertips and floor is measured by a scale fixed to the wall. Individual scores have a limited validity but repeated measures are an appropriate clinical indicator for the mobility of the spine.

#### Schober sign (SS)

The Schober Sign (SS) is another method to assess spinal mobility [47], originally developed by Schober [48]. In order to assess the Schober Sign two marks are made on the skin overlying the lumbo-sacral spine while the patient stands erect. The first mark is made at the first process of the sacrum, the second mark 10 cm cranially of the first mark. Next the patient is asked to bend forward as far as possible while the legs remain outstretched. The distance between the two marks is measured in centimeters. SS and FTF were assessed as secondary measures as an objective marker of spinal mobility. This is related to daily functioning. These measures complement the self-reported data from ODQ in a meaningful way (see also [49]).

#### Leg length difference (LLD)

Leg length difference was measured as an indicator of pelvic obliquity. The procedure of measuring leg length difference is described above (see Treatment). A line indicating the position of both legs is drawn connecting the calves of a patient lying on their back. Next the patient is asked to sit up with the legs outstretched. The shift between the two lines in this sitting position indicates the leg length difference.

#### Quality of life profile for the chronically ill (PLC)

The Quality of Life Profile for the Chronically Ill is a health-related quality of life inventory especially designed for patients with chronic conditions and validated in German [50]. It consists of 40 items and 6 subscales: physical functioning, ability to relax and enjoy life, positive affect, negative affect, social contact, and social integration. Scores of the 6 subscales can be summed to a total score. The inventory is well validated and frequently used in German-speaking countries.

#### Primary and secondary outcomes

We predefined changes at t2 for pain (VAS and Korff pain grade), and functional limitations (Oswestry Disability Score) as primary outcomes. Secondary outcomes were changes in quality of life (PLC), spinal mobility (SS and FTF) and leg length difference (as indicator of pelvic obliquity) at t2 and all changes at t3.

### Procedures

Patients contacted the study center and were screened for eligibility. Next, they were invited to the respective study center and a clinical examination regarding inclusion/exclusion criteria was performed by CS (Freiburg) or DS (Bad Oldesloe). Patients were informed about the study and gave written informed consent. Having consented, patients filled in the questionnaires. After baseline measurements were completed patients received their group assignment. Patients assigned to Medi-Taping received their first treatment immediately after the assignment and appointments were scheduled for the following 2 weeks. Medi-Taping treatments lasted approx. 15 min. Patients assigned to standard treatment received the booklet on low back pain and were connected with the respective physiotherapy center in order to schedule six sessions of physiotherapy of 20 min duration. Patients receiving pharmacotherapy for their back pain were allowed to continue with this treatment as they wished.

Patients returned to the study center 1 week after the end of treatment (t2), were assessed again clinically (LLD, SS, FTF) and filled in questionnaires (ODQ, PLC, VAS, Korff). The same assessment was repeated at t3, approx. three months after baseline. Clinical examinations at t2 and t3 were made by trained examiners who had not been interacting with the patients and who were blinded against treatment allocation. One examiner was used at each center. Both examiners were trained by the three therapists interacting with the patients (DS, CS, NW).

After follow-up measurements patients in the standard treatment arm were offered the opportunity to receive Medi-Taping therapy if they so wished, free of charge.

### Power analysis

There is no prior study for the estimation of an effect size of an approach combining manual therapy in order to reduce pelvic obliquity in combination with taping for CLBP. Thus, a clinical-pragmatic approach was taken. Based on reports of practitioners and patients a superiority effect of more than half a standard deviation was assumed. With an effect size of d = 0.6–0.7 and an objective to recruit at least 100 patients in total, power ranging from 70% (d = 0.6, α = .016, two-tailed, conservative correction for multiple testing) to 85% (d = 0.7, α = .016, two-tailed) was achieved.

### Randomization and allocation

Randomization was performed by using the random number generator of IBM SPSS 22 and allocation was blinded. Patients were randomized in blocks of 20. At first five Blocks of 20 were randomized at once. With ongoing recruitment, a sixth block of 20 patients was randomized. Finally, for the inclusion of the last patients a seventh block was randomized with only ten patients. Blocks were separated by study center. Study center North received 60 envelopes, South 70. The result of the randomization was printed on a result sheet that was sealed in an opaque envelope. The envelopes had consecutive numbers starting with one. The result sheet contained again the consecutive number, the group assignment and space to fill in date and time of opening, patient ID and name, name of person handing over the group assignment and their respective signature. Randomization was performed by SS who had no direct contact with patients otherwise. He ran the random number generator and sealed the envelopes that were then handed over to the respective clinician. The envelopes were opened in the presence of the respective patient in subsequent order. This process was documented by filling in the above result sheet.

### Blinding

All measurements at baseline took place before group assignment and were thus blinded. The patients themselves knew whether they received Medi-Taping or standard care and were thus not blinded. Measurements at t2 and t3 were performed by MDs who had no prior contact with the patients and who were blind to the group assignment. Patients were asked not to reveal any information regarding the therapy received to the examiner in order to maintain the blinding.

### Statistical methods

The study was evaluated according to the intention to treat approach. Missing data on questionnaire scales up to 20% of the total item number of the scale were replaced by means of the other items of the respective scale. All other missing data were replaced by regression-based imputations. Predictors for the imputation were the respective baseline value as well as age, gender, education, VAS at baseline, and chronic pain severity grade at baseline.

For the assessment of the primary and secondary outcome at post-treatment (t2) general linear models were applied with group and study center as dichotomous and baseline measure (t1) as continuous predictors. Data that followed a Poisson distribution (finger-to-floor distance, Schober sign, Korff grading, difference in leg length) were evaluated with a linear model using a Poisson distribution, with baseline measure as continuous predictor and group and center as categorical predictors. Data that were continuous but deviated from a normal distribution (Oswestry Disability Questionnaire) were log-transformed prior to analysis. Since three primary outcome variables were applied we applied a correction for multiple testing according to Holm, which uses alpha/3 for the first criterion, alpha/2 for the second, alpha/1 for the third criterion [51].

For the assessment of the follow-up data a repeated measurement ANOVA with t1, t2, and t3 as within-subject factor and *group assignment* and *study center* as between-subject factor was performed. Here the interaction term *time x group* was the focus of the analysis. Degrees of freedom were corrected for sphericity according to Greenhouse-Geisser. Regarding effect sizes we report *partial η*^*2*^ from the respective analyses. However, in order to compare our findings we also computed *Cohen’s d* for the changes from *baseline to end of therapy* (t1-t2) and from *baseline to follow-up* (t1-t3), by subtracting the means and dividing them to the mean of the respective standard deviations. All analyses were conducted with IBM SPSS 23 or Statistica V. 8.

## Results

### Recruitment and patient sample

**Fig. 1:**
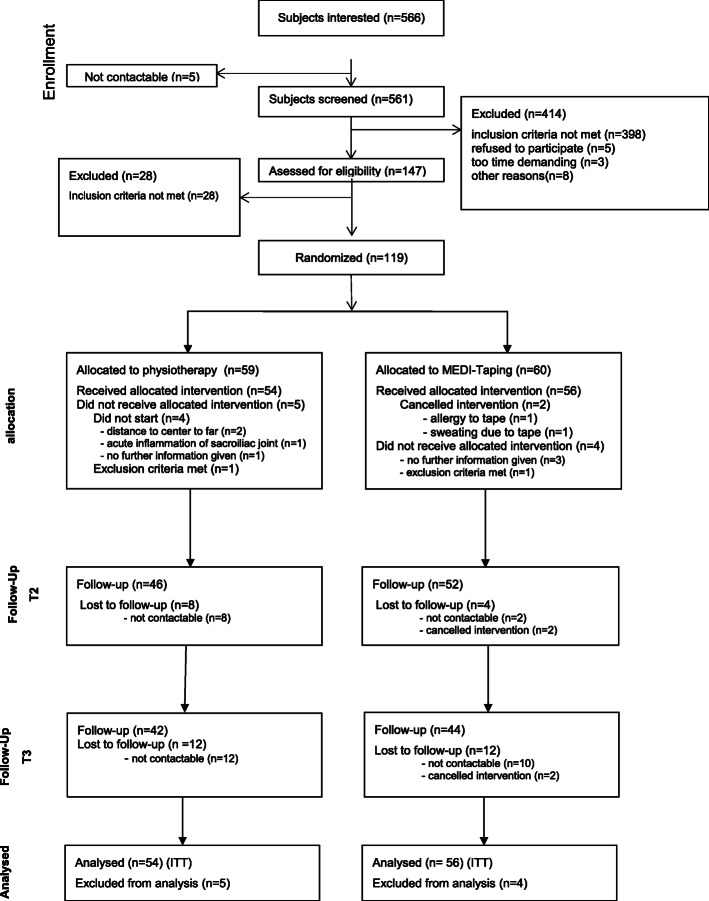
CONSORT patient flow

Recruitment took place between June 2015 and March 2016 and was terminated when the specified number of patients was reached. Overall, 561 patients were screened either per telephone or per email between June and October 2015. One hundred forty-seven patients were invited for a clinical examination, 119 patients showed up and finally 110 patients started with the intervention and were included into the intention to treat analysis. Of these 110 patients, 59 were treated at the study center South and 51 at the study center North. The exact patient flow can be seen in Fig. 1.

Table 1 presents the sociodemographic characteristics and the respective clinical baseline variables of the sample sorted by group. There were no significant baseline differences for any sociodemographic or clinical variable.

**Table 1:** Sociodemographic characteristics and clinical variables at baseline for the two groups; mean values (standard deviations)

	*Standard Care*	*Medi-Taping*
	*N* = 54	*N* = 56
Gender (% female)	68.5	69.6
Age (M;SD)	52.5 (13.63)	52.4 (12.81)
Weight	77.3 (16.80)	75.4 (18.58)
Height	171.5 (9.58)	171.6 (7.93)
Family status %		
married	63	69.6
divorced	14.8	8.9
single	11.1	16.1
living separate	7.4	3.6
Living %		
alone	22.6	23.2
With partner	66	71.4
Shared flat	7.5	5.4
With parents	3.8	0
Education %		
In education		
Basic schooling (9 years)	22.2	14.3
GSCE (10 years)	37	35.7
A-level (12–13 years)	40.7	50
Visual Analogue Score (pain)	5.66 (1.624)	5.17 (1.880)
Chronic Pain Grading	1.87 (0.702)	1.71 (0.756)
Oswestry Disability Q. (function)	41.20 (12.609)	42.24 (13.245)
Quality of Life (PLC)	15.32 (3.395)	15.65 (3.087)
Leg Length Difference	8.51 (5.657)	7.77 (5.271)
Finger-to-Floor Distance	8.86 (9.116)	6.67 (10.609)
Schober Sign	14.49 (1.065)	14.54 (1.416)

We also compared the baseline data for the two study centers South (*N* = 59) and North (*N* = 51). Samples were comparable for all variables except pain (VAS) and leg length difference (LLD), with the South sample reporting significantly more pain than the North one (South M = 5.79, SD = 1.4, North M = 4.97, SD = 2.0, T = 2.494, df = 108, *p* = .014). Leg length difference was smaller in the South center (M = 5.36, SD = 3.15) than in the North center (M = 11.34, SD = 5.8). This difference was highly significant (T = 6.847, df = 108, *p* < .001).

### Analyses of hypotheses

All analyses in the results section are based on the ITT-sample with *N* = 110 (Medi-Taping *n* = 56, standard care *n* = 54). Table 2 reports the descriptive data for all variables and all measurements by group. Tables 3 and 4 report the results of the appropriate linear models for main outcomes and and secondary outcomes. Here we find a significant difference for health-related quality of life (PLC) and for Finger-to-Floor Distance. Patients in the Medi-Taping group improved significantly more in quality of life than patients in the standard care group independent of study center. In Finger-to-Floor Distance patients in the Medi-Taping group had significantly better results, improving by 28% compared with control-group patients; here a center effect was obvious (RR = 1.12).

**Table 2:** Descriptive data for all clinical parameters for the two groups and the three measurement points baseline, post treatment, follow-up (3 months). Means (standard deviations), difference scores at post-treatment and follow-up [95% confidence intervals] difference scores between groups are based on changes from baseline to post or from baseline to follow-up, respectively

	Medi-Taping Means (SDs) (*n* = 56)			Standard Care Means (SDs) (*n* = 54)			Difference at Post-Treatment	Difference at Follow-up
	Baseline	Post	Follow-up	Baseline	Post	Follow-up	Means [95% CIs]^b^	Means [95% CIs]^b^
VAS – Visual Analogue Scale^a^	5.17 (1.88)	3.31 (2.32)	3.30 (2.17)	5.66 (1.62)	4.19 (2.07)	4.60 (2.24)	0.38 [−0.45; 1.21]	0.81 [−0.11; 1.73]
CPGS – Korff Pain Grading Scale^a^	1.71 (0.76)	1.61 (0.78)	1.36 (0.59)	1.87 (0.70)	1.57 (0.79)	1.56 (0.79)	-0.19 [−0.46; 0.08]	0.04 [−0.23; 0.32]
ODQ - Oswestry Disability quest.^a^	42.23 (13.24)	36.70 (14.42)	35.12 (12.47)	41.20 (12.61)	38.02 (14.26)	38.35 (13.47)	2.35 [−0.77; 5.48]	4.26 [0.43; 8.08]
PLC – Quality of Life Profile	15.65 (3.09)	17.16 (3.16)	16.97 (3.21)	15.32 (3.39)	15.71 (3.32)	15.65 (3.50)	1.12 [0.24;2.0]	0.99 [−0.15; 2.14]
LLD - Leg Length Difference (mm)	7.77 (5.27)	3.14 (3.89)	3.42 (3.15)	8.51 (5.66)	3.39 (3.17)	3.37 (3.36)	−0.5 [−2.62;1.63]	−0.8 [−2.9; 1.3]
FTF - Finger to Floor Distance (cm)	6.67 (10.61)	5.79 (10.15)	6.08 (9.60)	8.86 (9.12)	8.62 (8.88)	8.86 (8.74)	0.64 [−1.09; 2.37]	0.58 [−1.06; 2.22]
SS - Schober sign	14.54 (1.42)	13.99 (1.10)	14.31 (1.27)	14.49 (1.07)	14.15 (0.93)	14.39 (0.89)	0.21 [−0.18; 0.61]	0.13 [− 0.35; 0.61]

^a^primary outcomes at t2

^b^negative values for LLD and CPGS signal a higher benefit for the control group

**Table 3:** Results of linear models for the primary outcome variables pain (VAS), and functional limitations (ODQ) at post treatment (t2), and for secondary outcome (PLC) baseline values entered as covariates

*Variable*	*factor*	*F*	*df*	*p-value*	*part. η*^*2*^
VAS	group	2.718	1	.10	.025
	group x center	1.229	1	.27	.012
ODQ	group	2.12	1	.14	.02
	group x center	0.02	1	.88	.0002
PLC	group	8.564	1	.004**	.075
	group x center	0.146	1	.70	.001

** *p* < .01

**Table 4:** Results of linear models based on Poisson distribution for the primary outcome variable chronic pain grade severity according to CPGS (Korff) at post treatment (t2), and secondary outcomes (leg length difference, Schober sign, finger to floor distance) baseline values entered as covariates

*Variable*	*Effect*	*Wald Chi2*	*df*	*p-value*	*Estimate*	*Conf Int low*	*Conf Int high*	*Rate Ratio*
CPGS	group	0.20	1	.64	−0.03	−0.18	0.11	0.97
	group x center	0.04	1	.83	0.015	−0.13	0.16	1.01
LLD	group	0.75	1	.38	0.047	−0.059	0.154	1.048
	group x center	6.29	1	.012	0.136	0.03	0.24	1.145
Schober	group	0.09	1	.76	0.007	−0.04	0.05	1.007
	group x center	0.12	1	.72	−0.009	−0.06	0.04	0.991
FTF	group	45.97	1	<.00001	0.258	0.18	0.33	1.29
	group x center	10.02	1	.0015	0.115	0.044	0.187	1.12

*CPGS Korff Pain Grading Scale, LLD* leg length difference, *FTF* finger to  floor distance

The results of the linear models testing group differences at post-treatment (t2) for the three primary outcome variables can be seen in Tables 3 and 4. There were no significant group differences at this time point. We also report interaction terms for the study centers, which all proved to be not significant.

**Fig. 2:**
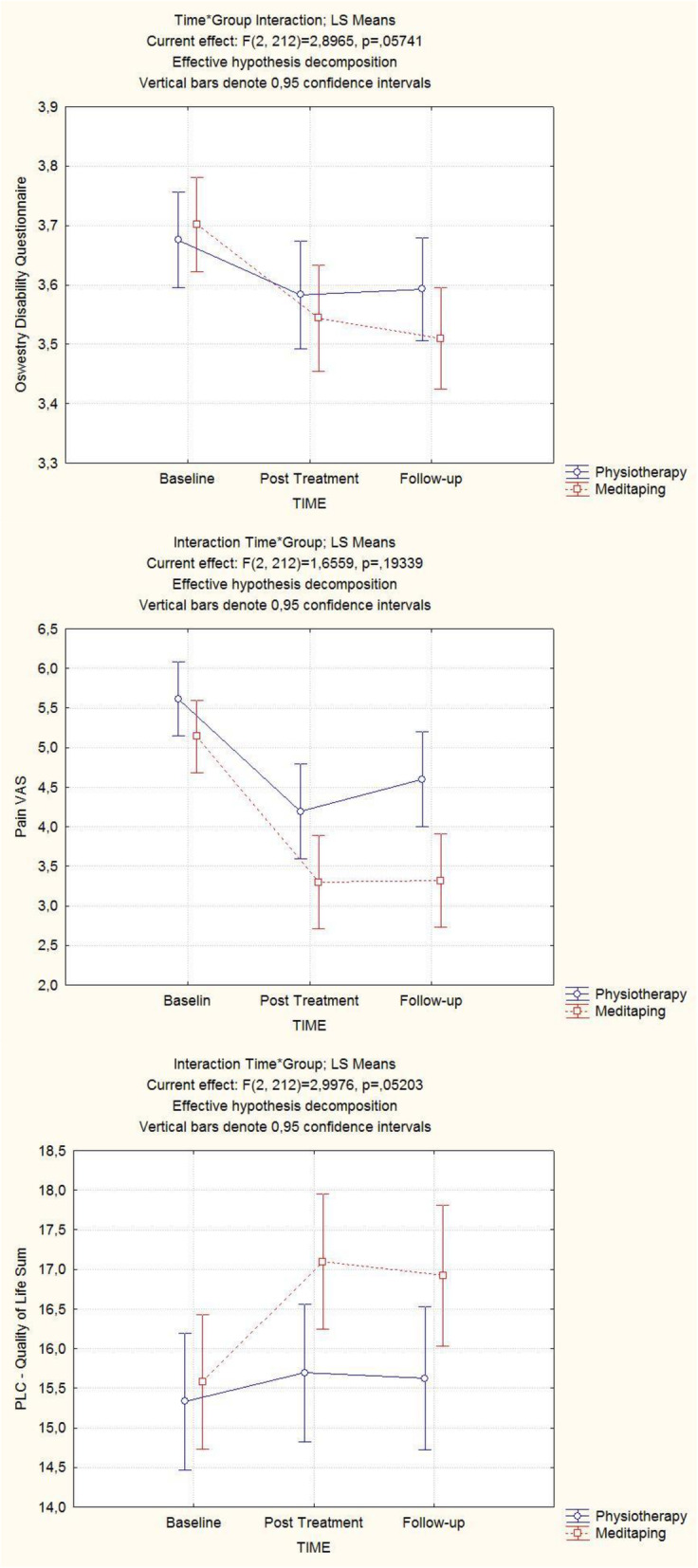
Interaction plots from RM-Anovas to illustrate the time course of Oswestry Disability Score (**a**), VAS (**b**), and Quality of Life (**c**)

For the assessment of the follow-up data we computed a repeated measurement ANCOVA over the post-treatment and follow-up measurement points with baseline as covariate. The results for both primary and secondary outcomes can be seen in Table 5. We report the relevant interaction terms for *time x group*. With VAS-Pain rating there was a significant interaction between *time* and *center *(F = 3.1, p_corrected_ = .048) indicating that patients in one center did better over time than in the other. No other interactions between *center *and *time *were detectable. Figure 2a-c illustrate the time course of the scores for VAS, Oswestry Disability Score and Quality of Life.

**Table 5:** Results of Repeated Measurement ANCOVA for follow-up data: interaction terms for group x time interaction with Greenhouse-Geisser corrected degrees of freedom and according *p*-values

	*F*	*corr. df*	*p*	*part. η*^*2*^
Visual Analogue Scale	1.656	1.972	.19	.015
Oswestry Disability Questionnaire	2.9	1.90	.060	.026
Quality of Life (PLC)	2.998	1.841	.057	.028

For the Poisson-distributed variables we computed Poisson-based linear models for the follow-up score as dependent variable with the baseline and post-treatment score as covariates and group and center as categorical predictors (Table 6).

**Table 6:** Results of Linear Models (Poisson distribution) for the secondary outcome variables; dependent variable score at follow-up; with baseline and post-treatment scores as covariate and group and center as factors

Variable	Effect	Wald Chi^2^	df	p-value	Estimate	Conf Int low	Conf Int high	Rate Ratio
LLD	group	0.17	1	.68	−0.02	−0.12	0.08	0.98
Schober	group	0.006	1	.93	0.007	−0.051	0.048	0.998
FTF	group	36,31	1	<.00001	0.222	0.15	0.29	1.25
CPGS	group	0.58	1	.44	0.06	−0.09	0.22	1.06

*LLD* Leg length difference, *FTF* finger-to-floor distance, *CPGS *Korff Pain Grading Scale

The interaction plots (Fig. 2a-c) illustrate that in each case the Medi-Taping group was better than the physiotherapy group and the improvements were more sustainable in the Medi-Taping group. We therefore conducted a multivariate analysis of these three variables together over both post-treatment and follow-up with baseline as a predictor as a kind of post-hoc exploratory analysis. Here, a clearly significant *time x group* interaction can be seen (Wilk’s lamba = 0.88; *p* = .004; partial eta^2^ = .12). This illustrates that our a-priori effect size assumption was too optimistic and the study suffered from a power problem.

### Clinical significance

With respect to the minimum clinically important differences (MCID) of 2.0 cm for VAS or 10 points for ODQ no effects of clinical significance were found between groups (at post-treatment: ODQ = 1.32, VAS = 0.88; at three-month follow-up: ODQ = 3.23, VAS = 1.30). Regarding within-group differences, pain (VAS) in the Medi-Taping group nearly reached the MCID from baseline to post treatment (1.86) and from baseline to three-month follow-up (1.87), but not for the difference from post-treatment to three-month follow-up (− 0.01). For the ODQ, the maximum change reached in within-group changes was 7.11 (Medi-Taping from baseline to three-month follow-up), from baseline to post-treatment the respective difference was 5.53, from post-treatment to three-month follow-up 1.58.

## Discussion

This pragmatic, examiner-blind randomized controlled trial of Medi-Taping versus physiotherapy in patients with chronic low back pain was the first of its kind. It was powered to detect medium-sized between-group effects in favor of Medi-Taping. All effects were pointing in the anticipated direction with patients in the Medi-Taping group doing better than controls. But our primary analysis failed to confirm this difference, as none of the primary outcomes showed a clearly statistically or clinically significant effect in favor of the experimental treatment. Some of the secondary outcome variables showed an effect: Quality of life improved significantly more in the Medi-Taping group with a small- to medium-sized effect of about 7% variance explained. And the finger-to-floor distance, which measures functionality, improved clearly and significantly more in the Medi-Taping group with a rate ratio of 1.29, indicating that a patient in the Medi-Taping group had on average a 29% better chance of improvement. This result was confirmed by the secondary analysis, a repeated measure analysis of variance of all variables, where the Oswestry Disability Score and the Quality of Life Score (PLC) just missed significance, and the Finger-to-Floor Distance was also highly significant. The trend illustrated by the interaction plots (Fig. 2a-c) and the multivariate analysis confirm that the Medi-Taping group is better in tendency, but the effect was much too small to be picked up by our study, and thus with respect to statistical significance the study suffered from a power problem. Regarding clinical significance, we could not demonstrate superiority of Medi-Taping with respect to pain (VAS) and function (ODQ). As regards to within-group differences, pain reduction in Medi-Taping patients came near the MCID but did not reach it.

Patients in both groups profited from the treatments, as is demonstrated by significant time effects of RM-ANOVAs and the within-group effect sizes. Thus, the strong control of the physiotherapy treatment was difficult to outrun with a trial of this size. An effect size of the magnitude found in our study (d = 0.37 for VAS at follow-up) would require 155 patients in each group to meet a power goal of 90% statistical power in a superiority trial. Thus, a fully powered trial would require more than 300 patients and would have to be three times as large as this trial.

The trial was pragmatic. Although all results provided by clinicians are blind, patients could not be blinded, and the taping treatment would not lend itself easily to any form of blinding control. Hence one might suspect reporting bias in the patient outcomes. However, as patients knew that they would be receiving one of two potentially effective treatments and clinicians were blinded, we believe that reporting bias was not a problem, especially since the difference between groups in self-reported measures was not larger than in clinician-reported outcomes. The patients in our study were certainly comparable to patients in general practice. They had a chronic problem. Their pain was partially disabling them in their function and it was of medium severity. Hence, our trial can be generalized to a community sample, and both treatments can be considered useful for chronic pain.

The most sensitive outcome proved to be finger-to-floor distance. This is a validated robust and objective measure of functionality [49]. For a follow-up study, this outcome should be kept, and the two other clinician-rated outcomes might be dropped. The patient-reported outcomes we used are standard. Only the quality of life scale really differentiated, while the Oswestry disability scale produced only small effects. This might be related to the fact that the patients in our sample were not severely compromised. Perhaps some other instrument, such as the Orebrö scale [52, 53], might be more useful and sensitive.

The intervention studied here is different from other effective chronic pain treatments. The physiotherapy offered here was a strong multimodal package with elements of mobilization and patient education. This is testified by the large pre-post effect sizes. There are almost no studies in the literature that compared kinesio taping with physiotherapy. Most studies provide physiotherapy to both groups and the experimental group then receives kinesio taping as add-on. In such comparisons, a recent review found an improvement in VAS of 0.62 and 5.15 points in the ODQ respectively [34].

### Our study had limitations

It was the first of its kind and hence the study was designed based on educated guesses and clinical impressions, which are notoriously unreliable, as documented by our over-optimistic power calculation. While we operated with two centers and thus had the benefit of a limited generalizability, ideally more centers should have been included. Perhaps a more realistic design would have been a non-inferiority trial, which, however, would require a considerably larger sample size [54, 55]. One of the centers was the clinic of DS who developed the Medi-Taping method and approached HW and SS to evaluate this form of treatment. This confounding of the roles might be a source of bias e.g. in assessing baseline values. On the other hand, all assessments at post-treatment and three-month follow-up were blinded and without the participation of DS. Furthermore, in all analyses, no differences could be found between the clinic of DS and the second center that operated independently at a University context and was located 800 km south of the former.

Another limitation was that we did not assess treatment adherence by the therapists. However, we had only three therapists, who were trained together and developed the treatment manual of the study during this training. In this context we are confident that there was only minimal variance in treatment delivery. Finally, our trial was only registered retrospectively. Thus, selection and reporting of outcomes and analyses cannot not be demonstrated to be prespecified. However, we submitted a detailed study protocol to the ethics committee before the start of the trial that contains all relevant details and can be obtained on request.

## Conclusions

We conclude from our pragmatic, partially-blinded randomized study that Medi-Taping, a purported way of correcting pelvic obliquity and chronic tension, is a treatment modality similar in effectiveness in treating chronic low back pain to a complex physiotherapy and patient education program with respect to pain, function and quality of life. There are indications that 2 months after the end of treatment Medi-Taping improves quality of life more than standard physiotherapy, but these indications are tentative and require replication in an adequately powered study.

## Data Availability

The datasets used and/or analyzed during the current study are available from the corresponding author on reasonable request.

## Abbreviations

ANOVA: Analysis of variance
ÄZQ: Ärztliches Zentrum für Qualität in der Medizin
CLBP: Chronic low-back pain
CNT: Coxib and traditional NSAID Trialists’ Collaboration
Conf Int: Confidence Interval
CPGS: Chronic Pain Grade Scale/Korff Grading
CPMP: Committee for proprietary medicinal products
DF: degrees of freedom
FTF: Fingertip to floor
GSCE: General Certificate of Secondary Education
LBP: Low-back pain
LLD: Leg length difference
LS Means: Least squares means
MCID: Minimum clinically important difference
MDs: Medical Doctors
ODQ: Oswestry low back pain disability questionnaire
PLC: Quality of Life Profile for the Chronically Ill
RCT: Randomized controlled trial
SS: Schober sign
VAS: Visual analog scale
